# Targeted Data Augmentation and Hierarchical Classification with Deep Learning for Fish Species Identification in Underwater Images

**DOI:** 10.3390/jimaging8080214

**Published:** 2022-08-01

**Authors:** Abdelouahid Ben Tamou, Abdesslam Benzinou, Kamal Nasreddine

**Affiliations:** 1 ENIB, UMR CNRS 6285 LabSTICC, 29238 Brest, France; abdelouahid.bentamou@univ-brest.fr (A.B.T.); nasreddine@enib.fr (K.N.); 2 Univ Brest, UMR CNRS 6285 LabSTICC, 29238 Brest, France

**Keywords:** underwater image, fish recognition, deep learning, convolutional neural network, hierarchical classification

## Abstract

In this paper, we address fish species identification in underwater video for marine monitoring applications such as the study of marine biodiversity. Video is the least disruptive monitoring method for fish but requires efficient techniques of image processing and analysis to overcome challenging underwater environments. We propose two Deep Convolutional Neural Network (CNN) approaches for fish species classification in unconstrained underwater environment. In the first approach, we use a traditional transfer learning framework and we investigate a new technique based on training/validation loss curves for targeted data augmentation. In the second approach, we propose a hierarchical CNN classification to classify fish first into family levels and then into species categories. To demonstrate the effectiveness of the proposed approaches, experiments are carried out on two benchmark datasets for automatic fish identification in unconstrained underwater environment. The proposed approaches yield accuracies of 99.86% and 81.53% on the Fish Recognition Ground-Truth dataset and LifeClef 2015 Fish dataset, respectively.

## 1. Introduction

Underwater camera systems are used extensively in scientific, industrial and military fields for exploring and studying underwater environments. They are useful for studying biodiversity and analyzing the interaction of animal species with their environment. They can also be used for observing the impact of human activities on the marine environment due to commercial overfishing [1] and industrial pollution. Moreover, these systems are nondestructive, do not perturb the environment and generate a large amount of visual data usable at any time. Several attempts have been made to automatically extract key information from these videos such as fish species [2,3,4,5,6,7,8], abundance [9] or behavior of the animal [10] in the video. This processing is an advantage compared with manual processing which is time-consuming, labor-intensive, costly, a relatively off-putting task and error prone, especially for species with similar appearances.

**Figure 1:**
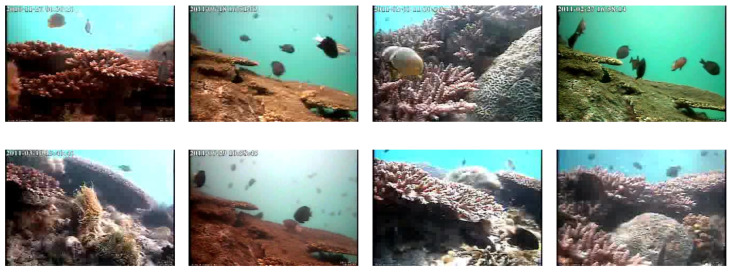
Examples of underwater images from different videos of the LifeClef 2015 Fish dataset. These examples illustrate the high variation in unconstrained underwater environments such as complex, crowded and dynamic backgrounds and luminosity variation.

However, automatic processing of underwater videos is not yet supplied due to the difficulties presented by the underwater environment which poses great challenges for computer vision (Figure 1). The luminosity changes frequently because of the ocean current, the visibility is limited and complex coral backgrounds sometimes change rapidly due to moving aquatic plants. Object recognition in underwater video images is an open challenge in pattern recognition, especially when dealing with fish species recognition. The fish move freely in all directions, they can also hide behind rocks and algae. In addition, the problems of fish overlapping and of the similarity in shape and patterns among fish of different species pose significant challenges in our application.

Automatic fish recognition involves two stages: (1) fish detection, which aims to detect and separate the fish from the background, and (2) fish classification, which aims to identify the species of each detected fish. In our previous work [11], we were interested to detect fish in unconstrained underwater videos. In this paper, we address the second stage in order to recognize live fish in underwater images.

Several works have been proposed in the last decade for automatic live fish identification using image processing techniques. The early works used hand-crafted features to recognize fish in open sea such as forward sequential feature selection (FSFS) [4], discriminant analysis approach [10], histogram of oriented gradients (HOG) [12] and SURF [13].

In the last few years, machine learning researchers focused on learned features by deep learning, especially with convolutional neural networks (CNNs), shown to be highly efficient in pattern recognition [14], object detection [15,16,17] and scene labeling [18] and language translation [19]. CNNs are able to extract high-level features from nonlinear data by transforming low-level input data through multiple levels of representation. CNNs have also shown high performance for underwater vision enhancement [20,21], and for fish detection [5,11,22,23]. For fish species classification, the first works based on CNNs applied well-known pretrained networks such as AlexNet [24,25,26], VGGNet [27], GoogleNet [28,29] and ResNet [30,31,32]. Our approaches use ResNeXt 101 network with 101 layers [33]. This architecture improves accuracy while reducing network complexity and the number of parameters by inheriting characteristics of ResNet, VGG and Inception (more details in Section 2.1). Other authors proposed their own architectures, but these are made up of only few convolutional layers, and hence are not really deep architectures [6,34,35]. In [7,8], the authors proposed hybrid deep architecture with hand-crafted techniques as principal component analysis (PCA), binary hashing and blockwise histograms to extract features of fish images. Some other works attempted to address fish detection and species classification within the same framework [5,22,36]. An interesting recent survey cites all works on fish species recognition [37,38].

In this work, we assume that a fish has already been detected in an underwater image, and we address fish identification by using CNNs. We present deep-learning-based approaches for fish species recognition in unconstrained underwater environment. As the underwater live fish datasets are of limited training data, it is not recommended to learn deep CNNs from scratch because of the huge number of parameters to be trained. To overcome the problem of limited underwater data, we use transfer learning [39] to retrain a pretrained network for coral reef fish classification in the open sea. The pretrained networks are trained on a large dataset as ImageNet [40]. There are many network topologies that have emerged such as AlexNet [14], VGG [41], GoogleNet [42], ResNet [43] and ResNeXt [33]. Furthermore, in the literature on deep-learning-based fish classification, we find methods that used data augmentation to improve the performances of the model and to avoid overfitting. However, they in general applied data augmentation on all fish training data even though the dataset is imbalanced [25]. Others have augmented the training data only for species whose sample number is less than a threshold in order to balance the dataset [7]. However, data augmentation requires more resources of memories and processors. Therefore, it could be necessary to proceed with data augmentation only for species that are difficult to be recognized. In this paper, we propose a new technique of targeted data augmentation based on training/validation loss curves.

In a traditional CNN-based multiclass classification task, the CNN model is designed to be sequential and generate a score for each class at the output. Then, the highest score determines the class of the query object. Thus, the CNN model treats all classes in the same way. With this “flat" classification structure, some classes could be misclassified more than others, especially for classes that have fewer examples or those which are difficult to be classified because of similarities with other classes. However, in fact, the property of general-to-specific category ordering often exists between classes [44], e.g., lion and tiger can usually be grouped as wild animals while bus and truck are vehicles. It is often easier to distinguish a lion from a bus than a tiger. This property indicates that the classification issue can be performed hierarchically instead of treating all classes as organized in a flat structure. In hierarchical classification, a classifier first classifies a lion in the coarse category of wild animals, then in the finer level as a lion. In this kind of classification, the error can be limited to a subcategory; this is an advantage of hierarchical classification because it makes the classification more informative than a traditional classification. For example, a classifier may confuse a lion with a tiger but knows that it should at least be a wild animal. On the other hand, in biology, there is a scientific classification of fish species based on taxonomy. This biological and hierarchical classification groups species with similarities and common characteristics in the same taxon. This biological classification inspires us to propose a hierarchical fish species classification based on CNN by first grouping species that have a common taxon in the same subset for an efficient classification.

For validation purposes, we carried out experiments on two underwater datasets: the Fish Recognition Ground-Truth (FRGT) (https://homepages.inf.ed.ac.uk/rbf/Fish4Knowledge/GROUNDTRUTH/RECOG/ accessed on 15 June 2022) and the LifeClef 2015 Fish (LCF-15) (www.imageclef.org/lifeclef/2015/fish accessed on 15 June 2022). The two underwater benchmark datasets are captured by underwater cameras in the open sea.

The main contributions of this paper are summarized as follows:

- We propose fish species hierarchical classification with deep CNN to classify fish, first by family and then by species, in unconstrained underwater images.
- We propose a new criterion for applying the data augmentation technique based on training/validation loss curves.
- The proposed schemes outperform state-of-the-art fish identification approaches on the FRGT and LCF-15 benchmark datasets.

The remainder is organized as follows. Section 2 presents the proposed approaches for underwater live fish classification. Section 3 describes the two benchmark datasets used in this work, gives the experimental results and performs a comparative study. Finally, the conclusion and perspectives are discussed in Section 4.

## 2. Proposed Approaches

Here, we propose two independent CNN approaches for efficient fish species classification. The first one, described in Section 2.1, is a flat classification where the CNN specifies the species of the input fish image at the output. This approach shows the contribution of transfer learning with targeted data augmentation on the accuracy. The second one, described in Section 2.2, is based on the property of general-to-specific category. It is a hierarchical fish species classification inspired from taxonomic fish classification. The CNN classifies each fish first into family level and then into species category.

### 2.1. CNN Transfer Learning and Targeted Data Augmentation

Currently, available labeled underwater live fish datasets are not large enough to train a CNN from scratch for fish species recognition. Moreover, immense resources of memories and processors are required. To overcome the difficulties imposed by limited fish training data, we used trained parameters of pretrained ResNeXt model [33] for the classification.

**Figure 2:**
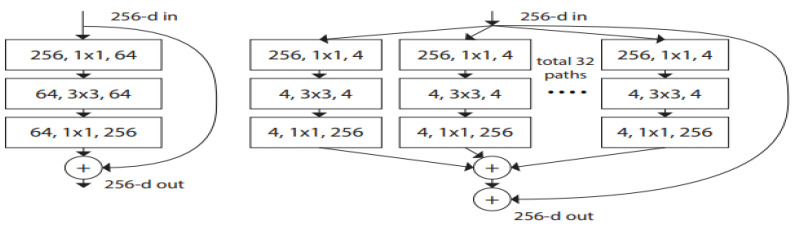
**(Left):** A building block of ResNet [43]. **(Right):** A block of ResNeXt with a given cardinality (here 32) [33]. A layer is shown as (# in channels, filter size, # out channels). These two blocks have roughly the same complexity.

ResNeXt [33] is a simple and highly modularized network architecture for image classification. The network inherits the key ideas of VGGNet [41], Inception [45] and ResNet [43]. It is constructed by repeating a building block (as in VGG) that aggregates a set of transforms (like Inception) while considering residual connections as ResNet. The suffix “Next” in ResNeXt means the next dimension (the size of the set of transformations) on top of the ResNet. This next dimension is called the “cardinality” dimension (Figure 2).

**Figure 3:**
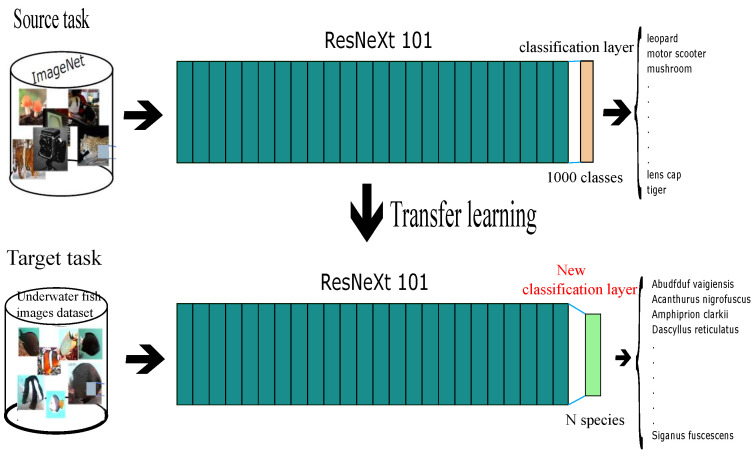
The proposed method for fish recognition based on transfer learning technique.

Figure 3 shows the global pipeline of our first proposed approach based on transfer learning using the ResNeXt 101 network as the pretrained model. We fine-tune ResNeXt by replacing the classification layer by a new one of N outputs corresponding to the number of species in our task. Then, we retrain only from scratch the new layer, and we keep the parameters of the earlier layers. For classification, the model applies the Softmax function on the last fully connected layer of the N-dimension. This function rescales the input vector $X=x_{1},\ldots ,x_{N}$ so that the elements of the N-dimensional output vector lie in the range [0,1] and sum to 1. It is defined for the *i*th element of *X* as:

(1) $$Softmax\left(x_{i}\right)=\frac{\exp\left(x_{i}\right)}{\sum_{j}^{N}\exp(x_{j})}$$

**Figure 4:**
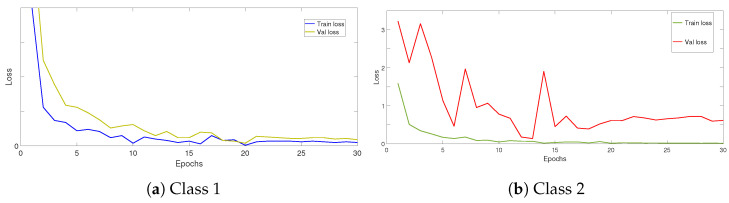
Example of training/validation loss curves: (**a**) well-converged, (**b**) badly converged.

On the other hand, among the proposed solutions to avoid the problem of limited training data and to improve the training of CNNs, there is a data augmentation technique that aims to artificially enlarge the training data. This technique is introduced to obtain better generalization by applying transformations on the existing data. In related works, some works applied data augmentation on all training data, even when the dataset is imbalanced. Others enlarged the training data for classes whose sample number is less than a threshold in order to balance the dataset. We propose, in this work, a new criterion for enlarging training data based on training and validation loss curves. First, we train the model on the whole dataset and calculate, in parallel, the loss for each class. Then, we inspect training and validation loss curves for each class. Then, we augment data only for classes whose loss curves are not converged. For example, Figure 4 illustrates two train/validation loss curves for two different classes. When the difference between train and validation loss curves is low, we do not augment data for this class (Figure 4a); on the other hand, we augment data only when the difference becomes higher (Figure 4b).

### 2.2. Hierarchical CNN Model for Fish Species Classification

In order to improve the performance of the model for fish species classification, we propose a new approach based on hierarchical classification inspired by the fish taxonomic classification. The model first classifies fish by family (coarse category) and then by species (finer category).

#### 2.2.1. Network Architecture

**Figure 5:**
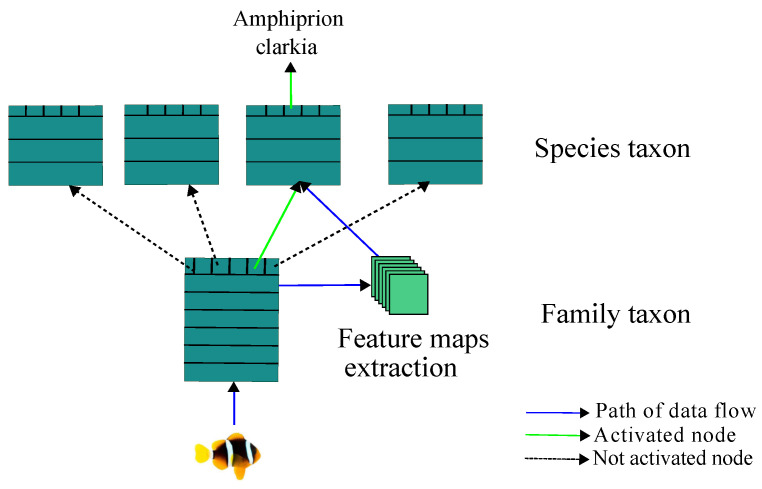
The proposed approach of Tree-CNN hierarchical classification for fish species recognition. The output of the root node is used to select the leaf node (family taxon) at the second level. Then, the leaf node classifies the fish in the species taxon.

The architecture of our proposed approach is composed of multiple nodes connected in a treelike manner [46] (Figure 5). The root node is the highest node of the tree. This node takes the fish image and generates feature maps. Then, it performs the first classification in order to classify the fish in a family. Following the result of this first classification, the feature maps are passed to the activated leaf node. This leaf node classifies the fish into species. Figure 5 shows the overall architecture of our model, which contains a root node and leaf nodes for a two-taxon classification network.

In this architecture, all nodes share common layers. These layers extract feature maps that feed the activated nodes. This network has several advantages, including the following:

1. The first layers of a CNN extract global features from the input image, while the higher ones extract more localized and class-specific features. Consequently, it is advantageous to share the lower layers because they are relevant to all classes.
2. The use of shared layers avoids replicating the same network multiple times in each node, which greatly reduces the computation time and the memory and processor resources. This allows the model to be used in real-time applications.
3. Layer sharing also reduces the number of parameters in the CNN, which speeds up the training of the model.
4. Leaf nodes are trained to be experts in the classification of species within the same family.

#### 2.2.2. Training Strategy

In order to build a CNN tree hierarchy, we group fish according to taxonomic fish classification. We use a top-down approach to train the hierarchy from the training set. As we embed nodes into the model, the number of parameters of the hierarchical architecture grows. This increases training complexity and the risk of overfitting. On the other hand, the imbalance of the training examples in a minibatch poses a major problem during the stochastic gradient descent as they could not be well routed to different leaf nodes. A large minibatch should be used to ensure that the parameter gradients in the leaf nodes are estimated by a sufficiently large number of training samples [47]. However, a large training minibatch increases the memory resources required and slows down the training process. Therefore, we address this problem by dividing the training into multiple stages instead of the whole training. We train the nodes in each level sequentially. Each node uses the cross-entropy loss that is defined as:

(2) $$l\left(x,y\right)=\frac{1}{M}\sum_{n=1}^{M}l_{n}$$

where *x* is the input, *y* is the target, *N* is the number of classes in the corresponding node and *M* spans the minibatch dimension, and $l_{n}$ is defined as:

(3) $$l_{n}=-\sum_{i=1}^{N}\log\frac{\exp(x_{n,i})}{\sum_{j=1}^{N}\exp\left(x_{n,j}\right)}y_{n,i}$$

**i**  **Root node training:**
  First, the root node is a pretrained CNN such as AlexNet [14], VGGNet [41], GoogleNet [42], ResNet [43], or ResNeXt 101 [33]. In this work, we use ResNeXt 101. It is efficient due to deeper layers and using the cardinality dimension, which make this network the 1st Runner Up of the ILSVRC 2017 classification task. So, we retrain ResNeXt on the examples of family taxon to extract the global features that are the input to each leaf node. This allows the leaf nodes to focus more on training the local features of each species. This root node uses the Softmax layer to learn the correlation between the input examples and classify them into the family taxon. At the end of this step, the parameters of the convolutional layers of this node are kept unchanged. The root node uses the same cross entropy loss defined in Equation (2) considering N the number of families in the dataset.
**ii**  **Leaf node training:**
  The leaf nodes can be trained independently in parallel. Each node should be specialized to classify the fish into a species taxon. All nodes are trained using the back-propagation algorithm. In the same way, all leaf nodes use cross-entropy loss (c.f Equation (2)) with *N* being the number of species in a corresponding family.
**iii**  **Test phase:**
  In the test phase, a query image is first forwarded to the root node where the Softmax layer produces a vector of scores indicating the probabilities that the image belongs to the families. The highest score determines the family node to which the given fish feature maps are routed. The leaf node assigns the species to the query image.

## 3. Results

We use two benchmark underwater datasets for evaluating the effectiveness of the proposed approaches for fish species classification. The two datasets contain images of fish of different colors, textures, positions, scales and orientations. Both are issued from the European project Fish4Knowledge (F4k) (www.fish4knowledge.eu accessed on 15 June 2022) [48]. During this project of five years, a large dataset of over 700,000 unconstrained underwater videos with more than 3000 fish species were collected in Taiwan, the largest fish biodiversity environment in the world.

We used a computer system equipped with an Intel Core-i5 processor with Geforce GTX 1050 Ti GPU and 2 Go GPU memory. We implemented the proposed approaches in Python using Pytorch.

In Section 3.1, we describe both benchmark datasets used in this paper. To evaluate the classification performances, we use the two measures presented in Section 3.2. Section 3.3 and Section 3.4 evaluate the proposed transfer learning and hierarchical classification approaches, respectively. Finally, we discuss and compare our results with the state-of-the-art methods in Section 3.5.

### 3.1. Benchmark Datasets

Here, we note that the input layer of ResNeXt requires an RGB image of size of 224×224×3. Therefore, we have to resize all input fish images to the same size for both benchmark experimental datasets.

#### 3.1.1. The Fish Recognition Ground-Truth (FRGT) Benchmark Dataset

**Figure 6:**
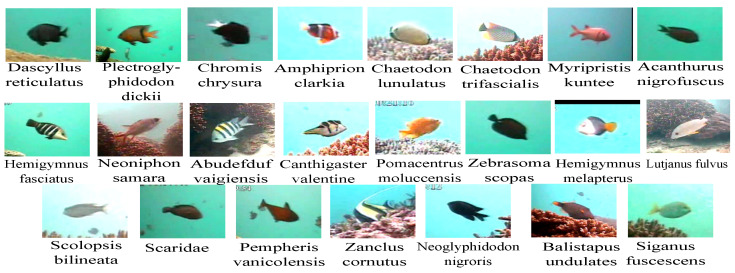
Sample images of 23 fish species in the FRGT dataset.

The FRGT dataset is an underwater live fish dataset that contains 27,370 fish images of 23 different species and their masks. The fish species are manually labeled by following instructions from marine biologists. Figure 6 shows examples of the 23 fish species and Table 1 gives the distribution of the fish species in the dataset. We note that the distribution of the dataset is imbalanced as the number of majority class instances is about 1000 times more than minority class instances. The fish images in this dataset have various sizes ranging from about 20 × 20 to about 200 × 200 pixels.

There is no test set in this dataset; in order to evaluate the performances of the proposed methods, we use 7-fold cross-validation as in [7,25]. The total images are divided into three subsets: 5/7 for training, 1/7 for validation and 1/7 for test. The final result is calculated as the average performance of the seven running times. As the number of different fish species is quite imbalanced, each class is divided in the same proportion randomly.

#### 3.1.2. LifeClef 2015 Fish (LCF-15) Benchmark Dataset

**Figure 7:**
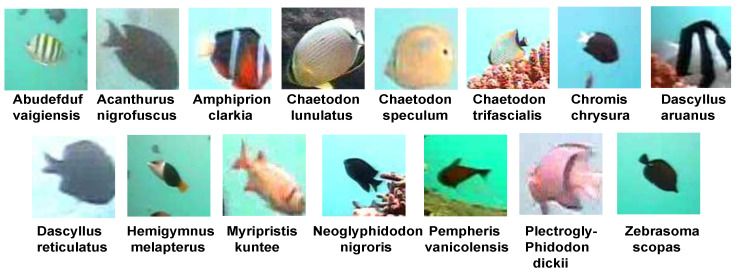
Sample images of 15 fish species in the LCF-15 dataset.

The LCF-15 is an underwater live fish dataset. The training set consists of 20 annotated videos and more than 22,000 annotated sample images. In this dataset, we have 15 different fish species. Figure 7 shows examples of the 15 fish species, and Table 2 gives the distribution of the fish species in the dataset. Each video is manually labeled and agreed on by two specialist experts. The dataset is imbalanced in the number of instances of different species; for example, the number of the species ‘Dascyllus reticulates’ is about 40 times more than the species ‘Chaetodon speculum’. Like the FRGT dataset, the fish images also have various sizes ranging from about 20 × 20 to about 200 × 200 pixels.

In experiments, we divide this training set into two subsets: 80% for training and 20% for validation (Table 2). The test set has 73 annotated videos. We note that for three fish species there are no occurrences in the test set (Table 2). This is conducted to evaluate the method’s capability to reject false positives. Compared with the first dataset, LCF-15 dataset provides challenging underwater videos marked by more noisy and blurry environments, complex and dynamic backgrounds and poor lighting conditions [34].

Finally, we note that fish images can be extracted from videos using the available ground-truth of fish bounding boxes.

### 3.2. Evaluation Metrics

We evaluate the effectiveness of both our approaches by adopting three measures: Precision of each class ($P_{i}$), where *i* is the index of class, Average Precision (AP) and Average Count or Accuracy (AC):

(4) $$P_{i}=\frac{TruePositive_{i}}{TruePositive_{i}+FalsePositive_{i}}$$

(5) $$AP=\frac{1}{N}\sum_{i=1}^{N}P_{i}$$

(6) $$AC=\frac{\sum_{i=1}^{N}TruePositive_{i}}{\sum_{i=1}^{N}\left(TruePositive_{i}+FalsePositive_{i}\right)}$$

where *N* refers to the number of dataset classes.

### 3.3. CNN Transfer Learning and Targeted Data Augmentation Approach

In this section, we evaluate the proposed approach of CNN transfer learning on both datasets with and without targeted data augmentation.

**Figure 8:**
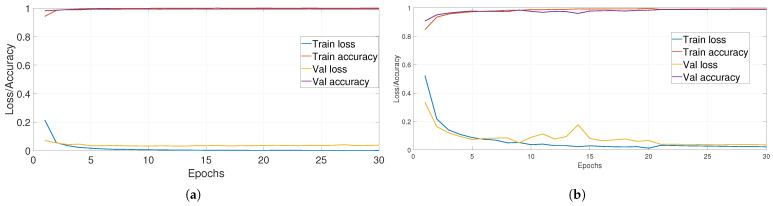
Training/validation loss curves: (**a**) on the FRGT dataset and (**b**) on the LCF-15 dataset.

**Figure 9:**
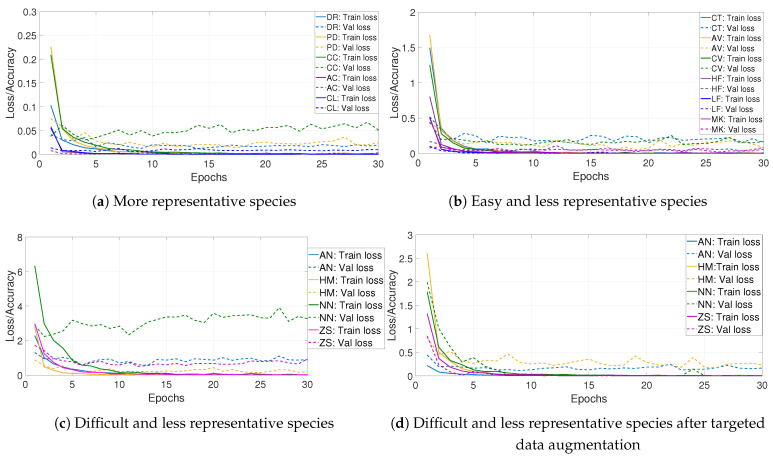
Training/validation loss curves for some species of the FRGT dataset. (**a**,**b**) show well converged curves for, respectively, species that are most representative and species that are less representative but easy to be recognized. (**c**) shows badly converged curves for species that are less representative and difficult to be recognized with available data. (**d**) illustrates the loss curves of species in (**c**) with targeted data augmentation.

First, we note that inspecting training/validation loss curves is not enough to conclude if the model has learned all classes well. Figure 8 illustrates the loss function per epochs of fine-tuning ResNeXt on FRGT (a) and LCF-15 (b) datasets. We can observe that the model has globally well converged and does not suffer from overfitting. In order to define classes that need to enlarge their training data, we inspect training/validation loss for each species, as illustrated in Figure 9 for the FRGT dataset.

Figure 9a–c and Table 3 show results of three categories of species into the FRGT dataset. The first category contains the more representative species (DR, PD, CC, AC and CL). Their loss curves have well converged. The second category contains some less representative species that are easy to be recognized (CT, MK, HF, NS, AV, CV, PM, LF, SB, S, PV, ZC and SF); the corresponding loss curves have well converged also. The third category has the species (AN, ZS, HM, NN and BU) that are less representative and difficult to be recognized due to the shape and color similarities to others species, especially for the species AN and NN, where 16.13% and 25% of test samples, respectively, are classified as DR. The training/validation loss curves for these species suffer from irregularities, especially for the species NN. Therefore, we only proceed by data augmentation for species of the third category, what we call targeted data augmentation. New loss curves are shown in Figure 9d and the precision of 23 fish species after data augmentation is given in Table 3.

**Figure 10:**
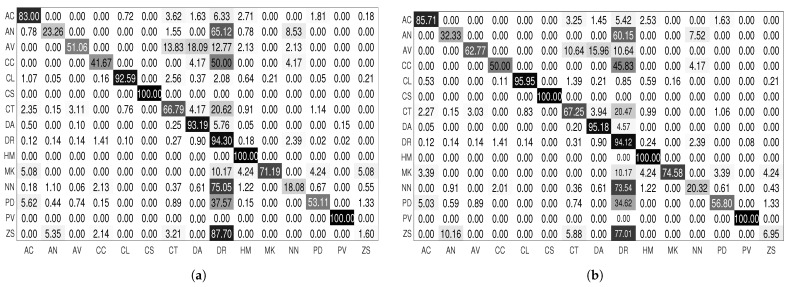
Confusion matrix of CNN transfer learning approach without (**a**) and with (**b**) targeted data augmentation for the LCF-15 dataset.

We proceed with the same technique for the LCF-15 dataset. We inspect training/validation loss for each species and we obtain only two categories: species with well-converged training/validation loss curves (AC, CL, CS, DA, DR, HM and PV) and species whose training/validation loss curves suffer from irregularities (AN, AV, CC, CT, MK, NN, PD and ZS). We proceed with data augmentation for the second category of species. Figure 10 illustrates the confusion matrix of our approach on the test set of the LCF-15 dataset before and after applying targeted data augmentation. Each row of the matrix represents the instances in a target class, while each column represents the instances in a predicted class.

To perform data augmentation, we proceed as follows. We flip each fish sample horizontally to simulate a new sample where a fish is swimming in the opposite direction; then, we scale each fish image to different scales (tinier and larger). We also crop the images by removing one quarter from each side to eliminate parts of background. Finally, we rotate the fish images with angles −20${}^{{}^{\circ}}$, −10${}^{{}^{\circ}}$, 10${}^{{}^{\circ}}$ and 20${}^{{}^{\circ}}$ for the invariant rotation fish recognition issue.

From the loss curves, after applying the targeted data augmentation technique (Figure 9d), we can observe that this technique reduces overfitting and improves generalization. As a result, the performances of the model were improved. For the FRGT dataset (Table 3), the precision was significantly improved for some difficult species: AN is improved by 14.31%, ZS by 2.86% and NN by 21.87%. For the LCF-15 dataset (Figure 10), the precision of some species are improved, such as for AN by 38.99%, AV by 22.93%, CC by 19.99% and NN by 12.39%.

Table 4 shows the effects of targeted data augmentation on the AC and AP measures on both datasets. With targeted data augmentation, we reach the highest accuracy and precision on both datasets. For the FRGT dataset, we achieve AC and AP of **99.86%** and **98.59%**. We have, respectively, AC and AP of **78.47%** and **69.46%** on the LCF-15 dataset.

We conclude that generic input image transformations such as flipping, cropping, scaling and rotation are helpful for reducing overfitting on some classes and may substantially improve generalization.

### 3.4. Hierarchical Classification Approach

We evaluate our hierarchical classification approach on the LCF-15 dataset, where accuracy was not high enough with CNN transfer learning.

**Figure 11:**
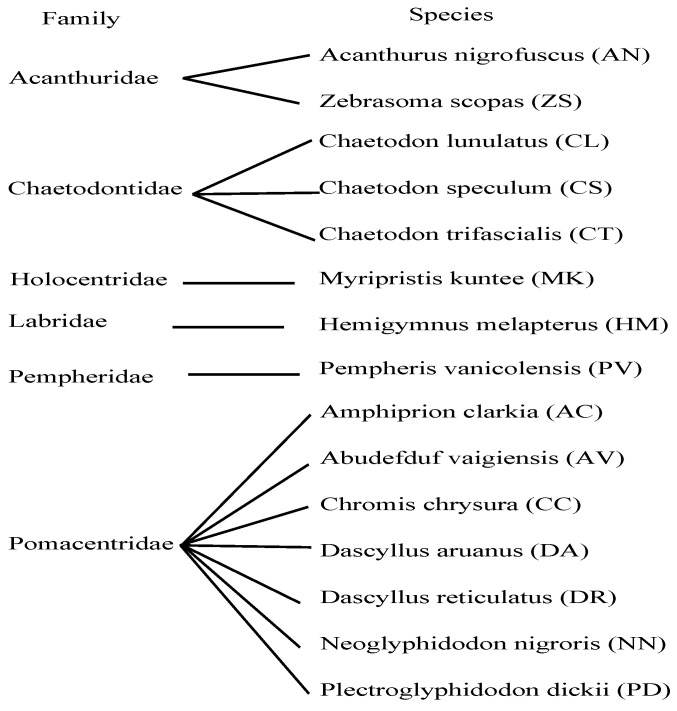
Taxonomic fish species classification of the LCF-15 images dataset.

Figure 11 illustrates the taxonomic fish classification of the LCF-15 dataset. We can see that the 15 species in this dataset can be grouped into 6 families. The Pomacentridae family is the largest one, it contains 7 species. The Chaetodontidae family contains 3 species and the Acanthuridae family contains 2 species. Finally, the Holocentridae, Labridae and Pempheridae families contain one species each. The root node therefore has 6 outputs corresponding to the 6 families.

**Figure 12:**
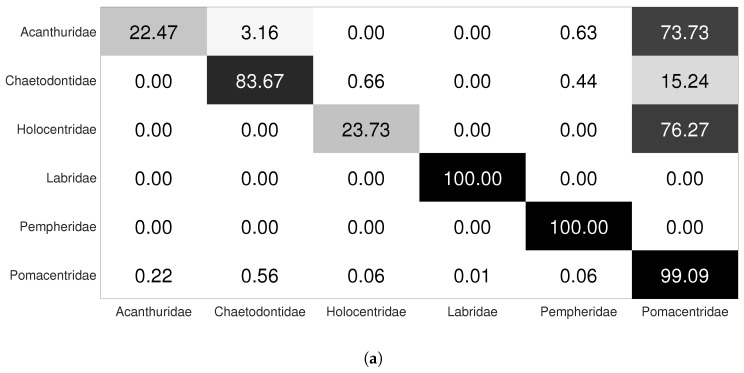

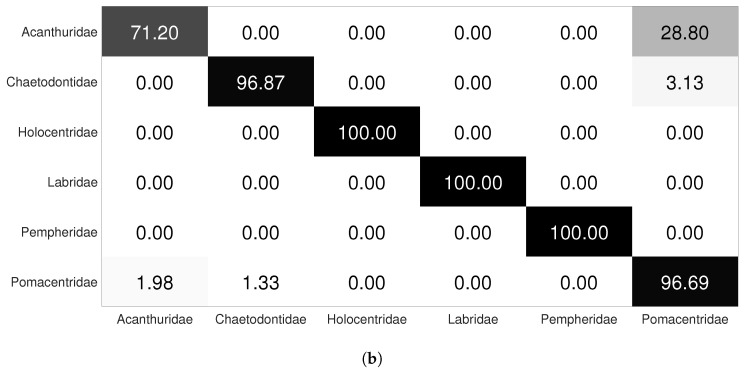
Root node confusion matrix for the LCF-15 dataset: (**a**) without data augmentation and (**b**) with targeted data augmentation.

In a hierarchical classification, the first nodes must perform well because if they misclassify the input images from the beginning, the child nodes will also misclassify them. Table 5 shows the performance of the root node of the hierarchical model for the LCF-15 dataset. The classifier of this node performs well, it achieves an accuracy of 92.98%. Figure 12a shows the confusion matrix of the root node without the use of data augmentation. According to the confusion matrix, most fish tend to be classified into the Pomacentridae family because the dataset is unbalanced and the Pomacentridae family is the most representative family.

As we said, the root node should perform well enough to reduce the classification error between families. To perform this, we increase the fish images, especially for the families that are difficult to be identified, namely the Acanthuridae, Chaetodontidae and Holocentridae families. We can see from Table 5 and Figure 12b, which represent the confusion matrix after applying targeted data augmentation, that the performance of the classifier is significantly improved for the difficult families. The classifier achieves a classification rate of 96.17%. We can also see that there is still some confusion between the Acanthuridae and Pomacentridae families and the Chaetodontidae and Pomacentridae families because of the similarity of shape and color between the species of these families. Finally, all fish of the Holocentridae, Labridae and Pempheridae families are well-classified. So, we have in this dataset a misclassification between some families. In order to improve the classification performance, we add a class ‘Others’ in the leaf nodes of the Acanthuridae, Chaetodontiade and Pomacentridae families. The class ‘Others’ contains fish samples from the Pomacentridae family for the nodes Acanthuridae and Chaetodontidae and contains fish samples from Acanthuridae and Chaetodontidae for the node Pomacentridae.

**Figure 13:**
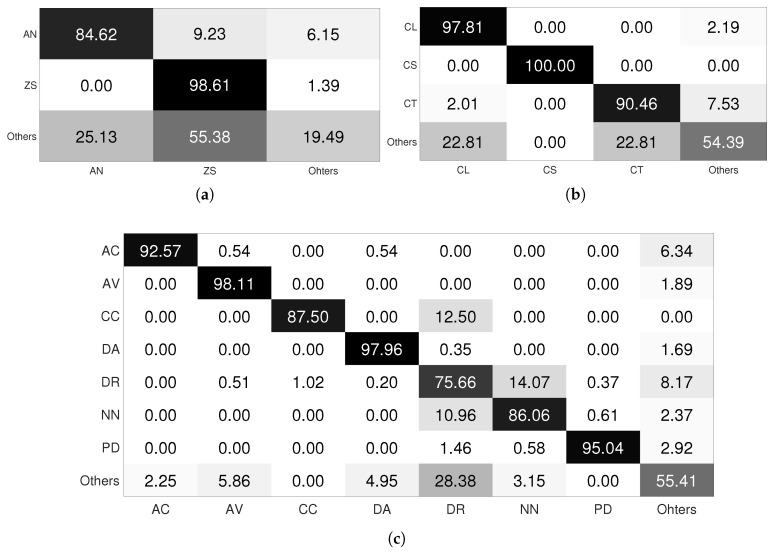
Leaf node confusion matrices for the LCF-15 dataset: (**a**) Acanthuridae node, (**b**) Chaetodontidae node and (**c**) Pomacentridae node.

The results are reported in Table 6. Figure 13 shows the confusion matrices for each leaf node. From Table 6 and Figure 13, we see that the performance of the Acanthuridae node is the worst, but looking at the confusion matrices of the nodes, we notice that the classifiers identify their own species very well but misclassify species of the ‘Others’ class.

**Figure 14:**
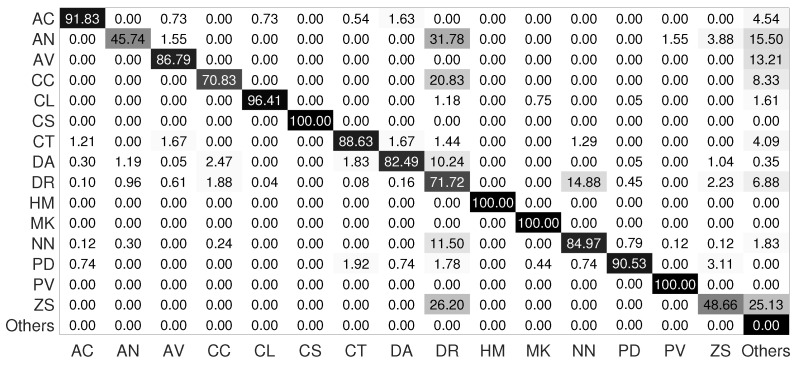
Confusion matrix of the hierarchical model for the LCF-15 dataset.

Finally, Figure 14 shows the confusion matrix of the whole hierarchical model. The hierarchical model achieves an accuracy of 81.53% (Table 6). According to the confusion matrix, the majority of the species are well-classified, including the difficult species. Therefore, we have significantly improved the classification performance compared with the flat classification, in which we obtained an accuracy of 78.47%.

### 3.5. Discussion and Comparative Study

Nowadays, underwater video systems are largely used by marine ecologists to study the biodiversity in underwater environments. Indeed, traditional techniques are destructive, affect fish behavior and demand time and labor costs. Many works proposed convolutional neural networks for fish species identification. However, these networks require large datasets due to the huge number of parameters to be trained, especially in deeper networks. In many real applications, there are not sufficient annotated training data, particularly in some specific tasks such as fish species identification. However, annotated training data is widely available for general computer vision tasks such as object recognition. Transferring network parameters learned on a general task to a specific task can solve the problem of lack of data, memory and processor resources, leading to better results.

In this study, we proposed to use the power of CNN transfer learning to recognize fish species in unconstrained underwater environments with low-resolution video images. Underwater environments are difficult and pose great challenges for computer vision due to luminosity condition, visibility, water turbidity and complex backgrounds together with the free movement of fish, fish overlapping and similarity in shape and patterns among fish.

Training a deep network from scratch with insufficient training data of low contrast and resolution undoubtedly results in poor performance. In [25], the authors trained the AlexNet architecture from scratch on the FRGT benchmark dataset, and they achieved a low AP value of 48.55%. Even with data augmentation, they reached an AP value of 61.54%, which is far from enough. Using transfer learning with data augmentation, they significantly improved the AP value to 99.64% [25]. This essentially shows the effectiveness of transfer learning for fish species recognition in underwater imagery. The most important contribution of our work is the high accuracy of fish species identification we reach. With only using transfer learning, we are able to achieve an AC value of **99.21%** on the FRGT benchmark dataset and an AC value of **76.89%** on LCF-15 benchmark dataset.

Data augmentation techniques aim to expand training data by applying some transformation methods on these data in order to improve the generalization and regularize the learning models. Transformation techniques include a range of image processing operations such as flipping, cropping, shifting, rotation and much more. This means we can simulate new and plausible fish samples at different scales, directions and luminosities. For example, the horizontal flip of fish samples produces a new fish sample swimming in the opposite direction. In [25], authors applied data augmentation on all training fish data, although the dataset is imbalanced. In [7], authors enlarged the training data for species whose sample number is fewer than 300. The latter solution may be not necessary for classes that are easy to be recognized. For these reasons, we proposed to inspect training and validation loss curves for each class to distinguish the difficult classes (Figure 9); we could differentiate between easy and difficult species. The loss curves for easy species have well converged without overfitting, while the loss curves for difficult species are not fully converged. So, applying data augmentation on these species can reduce overfitting and improve generalization. We reached an AC value of **99.86%** and **78.47%** on FRGT and LCF-15 datasets, respectively.

To overcome the problem of flat classification, one possible solution is to organize datasets hierarchically. In the fish recognition task, Huang et al. [4] proposed a Balance-Guaranteed Optimized Tree (BGOT) approach. This approach is designed to use the interclass similarities. BGOT uses hand-crafted methods to extract features and binary SVM classifiers and recursively chooses the best binary splitting by exhaustively searching all possible combinations. In our proposed CNN hierarchical classification approach, we divided the fish dataset hierarchically by using taxonomic fish classification. Our approach classifies fish at the root node into the family level and then into species categories at leaf nodes. Grouping fish that have similarities and common characteristics in the same subset then building a classifier for each subset improves classification performances. Indeed, the complicated task is divided in multiple easy subtasks. With this strategy, we improved the AC by 3.90% and AP by 20.79% on the LCF-15 dataset compared with the CNN transfer learning approach.

Table 7 and Table 8 show the comparison performances of our proposed approaches with the state-of-the-art methods on LCF-15 and FRGT datasets, respectively.

Szűcs et al. [13] used the sped-up robust features SURF method to extract features from fish images in order to feed an SVM classifier. Sun et al. [8] applied two deep architectures, PCANet [49] and NIN [50] to extract features from underwater images. A linear SVM classifier is used for classification. Iqbal et al. [27] proposed a modified AlexNet model. It is a reduced version of the AlexNet model comprised of four convolutional layers (instead of five convolutional layers) and two fully connected layers. The output of the first fully connected layer is of 9216 units instead of 4096 units as in the original AlexNet. Mathur et al. [31] fine-tuned the ResNet50 model by retraining only the last fully connected layers without any data augmentation. They used Adamx as the optimizer. We implemented the modified AlexNet and FishResNet models by using the same provided parameters in their papers. We also implemented the approach of Jalal et al. [22] based on the second branch of their hybrid approach, where they used YOLOv3 to detect and classify fish images. Zhang et al. [32] proposed AdvFish, which addresses the noisy background problem. They fine-tuned the ResNet50 model by adding a new term in the loss function. This term encourages the network to automatically differentiate the fish regions from the noisy background and pay more attention to the fish regions. Jäger et al. [24] used features extracted from the activations of the 7th hidden layer of the pretrained AlexNet model and fed a multiclass SVM classifier.

From Table 7, we observe that approaches based on deep learning perform better than the hand-crafted method. We can also see that CNN trained without fine-tuning is not efficient (CNN-SVM [24]: 66%) compared with CNNs trained with transfer learning (ResNeXt **78.47%**). Our proposed hierarchical classification outperforms the-state-of-the-art methods. These results confirm the challenging nature of this benchmark dataset that is marked by highly blurry images with background confusion with fish and higher degradation in terms of light intensity. We note that Salman et al. [34] reached an accuracy of 93.65% by testing their model on 7500 fish images issued from the LCF-15 dataset, but these fish images are not from the original test set provided in the dataset. At the moment that they tested their method, they did not use original training or the test split provided in the LCF-15 dataset. Moreover, Jalal et al. [22] did not use the original test set provided in the LCF-15 benchmark dataset. Instead, they merged the training and test sets, then they took 70% of the samples for training and 30% for testing. The test set of the LCF-15 benchmark dataset is highly blurry compared with the training set, which explains their high accuracy in their article compared with ours.

From Table 8, in Deep-CNN [6], a CNN with three convolutional layers is created and trained from scratch. In DeepFish [7], the same authors have eliminated the background by using available fish masks and trained the deep network with some hand-crafted layers that contain PCA and blockwise histograms to improve the species recognition performance. However, they marginally improved the accuracy by 0.07%, even with data augmentation. In AlexNet-Dir [25], the authors trained the AlexNet model directly with the underwater images without transferring any knowledge and without using data augmentation. They achieved a low AP value of 48.55%. In AlexNet-Soft [25], authors fine-tuned AlexNet with data augmentation including horizontal mirroring, cropping, subsampling and affine transformation; they achieved an AP value of 97.10%. Then, in AlexNet-SVM, they extracted feature maps with a retrained AlexNet in order to feed an SVM classifier; they achieved the best AP of 99.64%. We tested the approach of FishResNet [31] by using the same test set, we obtained 95.62%. We used the provided code in AdvFish [32], and we trained ResNet50 by using 7-Fold cross-validation; we achieved 90.99%. In our work, without data augmentation, we obtained an AC value of **99.21%** and AP value of **95.38%**. With targeted data augmentation only applied to species that are difficult to be recognized, and without using any other classifier, we obtained an AC of **99.86%** and AP of **98.59%**. We can also conclude that networks that are trained with transfer learning give better results than networks trained from scratch.

An important remark is that with our targeted data augmentation approach precision for others species, especially less representative species, are improved (CT, MK, HF, NS, AV, CV, S, PV and ZC) or unchanged (PM, LF, SB and SF), even though we did not apply data augmentation on these species. Additionally, they are higher compared with sate-of-the-art methods. We note that some methods have good accuracy, but looking at the species precision, we find that they are low for some species such as ResNet [30] for species DR, CC and AC, FishResNet [31] for species AN, ZS and NN, and DeepFish [7] for species AN and NN. We can conclude that our approach has better advantages compared with other methods. The targeted data augmentation technique significantly leads to improved precision for difficult species without downgrading precision for others classes.

## 4. Conclusions

In this paper, we presented two CNN approaches for the live fish recognition task in an unconstrained underwater environment. First, we proposed to transfer knowledge from the pretrained ResNeXt 101 network and retrained it on underwater fish image datasets. We have shown that the fine-tuned ResNeXt improves the model performances. We also analyzed the effects of data augmentation. As well, we have not augmented the data uniformly for all classes, however, we have proposed a targeted data augmentation technique based on training/validation loss curves for better performance. Second, inspired from taxonomic fish classification, we proposed a hierarchical classification that classifies fish first by family then by species. Experiments on two underwater live fish benchmark datasets, namely the Fish Recognition Ground-Truth dataset and LifeClef 2015 Fish dataset, demonstrated that our proposed approaches outperform various state-of-the-art methods for fish species identification and improved our previous results.

Our future work will aim to construct a fully automatic system that combines fish detection with species classification in the presence of a very large number of fish species.

## Figures and Tables

**Table 1:** The fish species distribution in the FRGT dataset.

ID	Species	Samples	ID	Species	Samples
DR	Dascyllus reticulatus	12,112	PM	Pomacentrus moluccensis	181
PD	Plectroglyphidodon dickii	2683	ZS	Zebrasoma scopas	90
CC	Chromis chrysura	3593	HM	Hemigymnus melapterus	42
AC	Amphiprion clarkia	4049	LF	Lutjanus fulvus	206
CL	Chaetodon lunulatus	2534	SB	Scolopsis bilineata	49
CT	Chaetodon trifascialis	190	S	Scaridae	56
MK	Myripristis kuntee	450	PV	Pempheris vanicolensis	29
AN	Acanthurus nigrofuscus	218	ZC	Zanclus cornutus	21
HF	Hemigymnus fasciatus	241	NN	Neoglyphidodon nigroris	16
NS	Neoniphon samara	299	BU	Balistapus undulates	41
AV	Abudefduf vaigiensis	98	SF	Siganus fuscescens	25
CV	Canthigaster valentine	147		Total	27,370

**Table 2:** The fish species distribution in the LCF-15 dataset.

ID	Species	Family	Training Set Size	Validation Set Size	Test Set Size
AN	Acanthurus nigrofuscus	Acanthuridae	2244	561	129
ZS	Zebrasoma scopas		274	69	187
CL	Chaetodon lunulatus	Chaetodontidae	2969	742	1876
CS	Chaetodon speculum		130	32	0
CT	Chaetodon trifascialis		545	136	1319
MK	Myripristis kuntee	Holocentridae	2597	649	118
HM	Hemigymnus melapterus	Labridae	285	71	0
PV	Pempheris Vanicolensis	Pempheridae	838	210	0
AC	Amphiprion clarkia	Pomacentridae	2677	669	553
AV	Abudefduf vaigiensis		349	87	94
CC	Chromis chrysura		3086	772	24
DA	Dascyllus aruanus		1422	355	2013
DR	Dascyllus reticulatus		5066	1267	4898
NN	Neoglyphidodon nigroris		171	43	1643
PD	Plectrogly-Phidodon dickii		2355	589	676
Total			25,008	6252	13,530

**Table 3:** Precision for 23 fish species of the FRGT dataset using CNN transfer learning approach without and with targeted data augmentation.

Species ID	Precision without Data Augmentation	Precision with Targeted Data Augmentation
	(%)	(%)
DR	99.83	99.91
PD	99.40	99.81
CC	99.22	99.89
AC	99.90	100
CL	99.83	100
CT	98.25	99.47
MK	98.81	99.78
AN	83.87	95.87
HF	98.77	100
NS	99.01	100
AV	97.06	100
CV	97.56	99.32
PM	100	100
ZS	96	98.75
HM	90.91	97.62
LF	100	100
SB	100	100
S	94.12	100
PV	92.86	100
ZC	92.31	100
NN	66.67	81.25
BU	93.33	100
SF	96	96

**Table 4:** Performance comparison with and without targeted data augmentation on the FRGT and LCF-15 datasets.

	Available	Data	Targeted	Data	Augmentation
Dataset	AC(%)	AP(%)	AC(%)		AP(%)
FRGT	99.21	95.38	99.86		98.59
LCF-15	76.89	65.99	78.47		69.46

**Table 5:** Performance of the root node of the hierarchical model for the LCF-15 dataset.

Node	AC (%)	AP (%)
Root node with available data	92.98	92.05
Root node with targeted data augmentation	96.17	94.13

**Table 6:** Performance of leaf nodes (species) in the hierarchical model for the LCF-15 dataset. Leaf nodes contain an additional class called ‘Others’.

Node	AC (%)	AP (%)
Acanthuridae	58.17	67.57
Chaetodontidae	94.66	85.67
Pomacentridae	83.75	86.04
Whole model	81.53	83.90

**Table 7:** Comparison of fish recognition accuracies of various methods on the LCF-15 dataset.

Approaches			Methods	AC(%)
Hand-crafted			SURF-SVM [13]	51
Deep learning	Without transfer learning	Feature extraction	PCANET-SVM [8]	77.27
		Training from scratch	Modified AlexNet [27]	72.25
	With transfer learning	Fine-tuning	FishResNet [31]	54.24
			Yolov3 [22]	72.63
			AdvFish [32]	74.54
			**ResNeXt (ours)**	**78.47**
		Feature extraction	CNN-SVM [24]	66
			NIN-SVM [8]	69.84
		Hierarchical	**ResNeXt (ours)**	**81.53**

**Table 8:** Comparison of fish recognition performances of various methods on the FRGT dataset.

Species	With Transfer Learning							Training from Scratch		
	Without Data Augmentation			With Data Augmentation				Without Data Augmentation		With Data Augmentation
	ResNeXt (Ours)	ResNet [30]	FishResNet [31]	ResNeXt (Ours)	AlexNet-Soft [25]	AlexNet-SVM [25]	AdvFish [32]	AlexNet-Dir [25]	Deep-CNN [6]	Deep-Fish [7]
DR	99.83	28	96.70	99.91	99.78	100	99.77	95.12	-	92.25
PD	99.40	92	94.36	99.81	98.79	99.77	99.74	41.32	-	97.39
CC	99.22	58	92.75	99.89	99.75	99.60	98.83	81.42	-	98.24
AC	99.90	65	98.72	100	99.97	100	99.83	92.44	-	100
CL	99.83	83	98.73	100	100	100	99.45	95.15	-	100
CT	98.25	100	75	99.47	100	99.38	93.75	52.83	-	96.30
MK	98.81	100	93.77	99.78	100	100	64.52	84.55	-	100
AN	83.87	86	53.20	95.87	89.05	96.41	95.24	11.81	-	67.74
HF	98.77	100	91.86	100	98.15	100	87.50	62.03	-	100
NS	99.01	100	100	100	100	100	78.57	100	-	100
AV	97.06	100	75.71	100	100	100	88.23	63.16	-	92.86
CV	97.56	100	93.33	99.32	100	100	71.43	43.75	-	95.24
PM	100	100	94.58	100	96.09	100	93.02	48.95	-	100
ZS	96.00	100	51.56	98.75	85.06	100	75.00	8.12	-	84.62
HM	90.91	100	66.66	97.62	100	100	76.92	47.37	-	66.67
LF	100	100	97.96	100	100	100	95.24	0	-	96.55
SB	100	100	82.86	100	100	100	100	14.29	-	85.71
S	94.12	100	92.50	100	86.67	96.56	100	33.33	-	100
PV	92.86	100	95.24	100	100	100	83.00	13.89	-	100
ZC	92.31	100	73.33	100	100	100	100	33.33	-	100
NN	66.67	100	37.50	81.25	84.62	100	75.00	85.71	-	50
BU	93.33	100	72.41	100	95.45	100	100	8.03	-	83.33
SF	96	100	83.33	96	100	100	83.00	0	-	100
AP	95.38	92	83.13	98.59	97.10	**99.64**	89.48	48.55	-	91.91
AC	99.21	98.03	95.46	**99.86**	-	-	98.16	-	98.57	98.64

## Data Availability

The Fish Recognition Ground-Truth dataset (https://homepages.inf.ed.ac.uk/rbf/Fish4Knowledge/GROUNDTRUTH/RECOG/ accessed on 15 June 2022) and the LifeClef 2015 Fish dataset (www.imageclef.org/lifeclef/2015/fish accessed on 15 June 2022).
